# Diagnostic Symptoms of Dislocation of the Head of the Femur

**Published:** 1829-01-01

**Authors:** 


### LVIII.

### DIAGNOSTIC SYMPTOMS OF DISLOCATION OF THE HEAD OF THE FEMUR INTO THE ISCHIATIC NOTCH. By Dr. BUCHANAN.*

Sir Astley Cooper has observed, that "of all the dislocations of the thigh, this is the most difficult to detect, because the length of the limb is but little altered, and the change in the position of the knee and foot is not so marked as in the dislocation upwards." The symptoms which Sir Astley details as diagnostic are the following.

"The limb is from half an inch to one inch shorter than the sound one, but rarely more than half an inch. The natural projection formed by the trochanter major is diminished, and is inclined towards the acetabulum, but still remains at right angles with the ilium. The head of the bone can only be felt in very thin persons, and then not very distinctly. The knee and foot are turned inwards, and the great toe rests against the ball of the great toe of the sound limb. When the patient is erect, the toe touches the ground, but the heel does not quite reach it, and the knee is bent, and projects a little forwards. The motions of the joint are, in a great degree, prevented, admitting of but slight flexion and rotation."

These symytoms, Dr. Buchanan considers as characteristic of dislocation, when it actually exists, but simulated so far by other affections of the joint, as to lead to error when adopted in every instance, as our guide. A truly diagnostic sign, then, being wanted, the author of the paper believes that he has found it, and proceeds to make it known. In the Summer of 1821, a young woman was brought into the Glasgow Infirmary, after falling from a height, a good deal bruised and affected, soon afterwards, with paralysis of the right leg. These symptoms had nearly disappeared when Dr. Buchanan

* *Glasgow Medical Journal, No.* IV.

first saw the case, but others were present closely corresponding with those laid down by Sir Astley Cooper, as marking dislocation into the ischiatic notch; an accident which was thought to have happened to the patient. Gradually, however, the limb re-assumed its natural aspect, and shewed that the doctors had been taken in.

"In reflecting upon the preceding case, it occurred to me, that it would be of much importance in supposed cases of dislocation into the ischiatic notch, to institute a comparison of the limbs, not while resting in the same plane with the body, but when bent to a right angle with the abdomen; the reason of which must be sufficiently apparent, from considering the anatomy of the joint, and the nature of the injury. The head of the femur being thrown almost directly backward, and very little upward, it is clear, that so long as the limbs remain in the plane of the body, there can be very little difference in their relative length; since that difference is only measured by the extent of the displacement upwards. But if the limbs be slowly bent towards the abdomen, the difference in their length must become greater and greater, till it attain a maximum, when the limbs are at right angles with the body; the luxated limb being then shortened by the whole extent of the dislocation backward. I had no opportunity of verifying this reasoning by actual observation, till the occurrence of the following case, in 1826, and I had then the satisfaction to find, that the mode of examination described above, yielded the only symptoms, which were perfectly unequivocal."

A child, three years of age, fell from a chair, and when seen by Dr. B. six days after the accident, presented the following appearance. The girl could not walk nor stand without assistance, but, when supported by her mother on the sound limb, the toes alone of the other touched the ground, were a little turned in, and rested in the same transverse line with the metatarsal bones of the opposite foot. The knee was considerably bent, and advanced before its fellow; the shortening of the limb, if any existed, could not have been considerable; the thigh could be bent towards the abdomen with facility, and seemingly without producing much pain, whilst abduction and rotation were not only painful, but comparatively difficult; the head of the femur could not be discovered; the trochanter was little displaced; the contour of the haunch not materially altered; and, lastly, the limb altogether had more the appearance of being drawn up by a voluntary effort, than of being actually shortened by displacement. These symptoms were dubious, but uncertainty vanished the instant the patient was placed upon her back upon a table, and both thighs bent to a right angle with the trunk of the body. The shortening immediately became most decided, the affected knee, resting in the hollow below the condyles of the opposite limb. The trochanter, also, was felt much farther back than on the sound side.

Dr. Watson, Dr. Young, and Dr. Auchincloss, being also satisfied of the nature of the accident, the reduction was effected next day, by placing the patient in a sitting position on a table, to which she was fastened down by a sheet, and secured by a man standing astride it behind her—performing extension by a bandage round the ankle—and lifting the head of the femur over the edge of the acetabulum, by means of a handkerchief passed under the thigh. The reduction took place with an audible snap.

This mode of examination, our author considers applicable to dislocation into the foramen ovale, but there is just this slight objection which strikes us, namely, that one of the distinguishing marks of these dislocations is the immobility, at any rate impaired mobility, of the limb. In the dislocation into the ischiatic notch, Sir Astley Cooper expressly states, that the thigh admits of but "slight flexion and rotation," and when the displacement is into the foramen ovale, we should think the want of motion would be greater still. In some cases, however, the one for example detailed by Dr. B., the limb was so mobile, as to admit of the application of the test, and, in such, it may prove a valuable addition to our diagnostic marks. In cases of contusion, or inflammation of the joint, simulating dislocation, such an examination seems perfectly applicable.

